# 2-Methyl-7-nitro-2,3-dihydro-1-benzofuran

**DOI:** 10.1107/S1600536808017728

**Published:** 2008-06-19

**Authors:** Mei-Li Feng, Yu-Feng Li, Shan Liu, Hai-Yu Yang, Hong-Jun Zhu

**Affiliations:** a Department of Applied Chemistry, College of Science, Nanjing University of Technology, Nanjing 210009, People’s Republic of China

## Abstract

The dihydro­furan ring of the title compound, C_9_H_9_NO_3_, adopts an envelope conformation. The nitro group is twisted slightly away from the attached benzene ring [dihedral angle = 21.9 (1)°].

## Related literature

For bond-length data, see: Allen *et al.* (1987 ▶). For details of the synthesis, see: Majumdar *et al.* (2008 ▶).
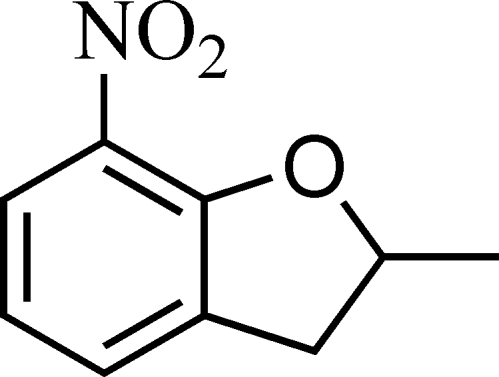


## Experimental

### 

#### Crystal data

C_9_H_9_NO_3_
*M* *_r_* = 179.17
Orthorhombic, 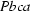

*a* = 8.4250 (17) Å
*b* = 7.2260 (14) Å
*c* = 28.295 (6) Å
*V* = 1722.6 (6) Å^3^
*Z* = 8
Mo *K*α radiation
μ = 0.10 mm^−1^
*T* = 298 (2) K
0.30 × 0.20 × 0.20 mm

#### Data collection

Enraf–Nonius CAD-4 diffractometer
Absorption correction: ψ scan (North *et al.*, 1968 ▶) *T*_min_ = 0.969, *T*_max_ = 0.979
2977 measured reflections
1551 independent reflections
829 reflections with *I* > 2σ(*I*)
*R*_int_ = 0.048
3 standard reflections every 200 reflections intensity decay: none

#### Refinement

*R*[*F* ^2^ > 2σ(*F* ^2^)] = 0.066
*wR*(*F* ^2^) = 0.182
*S* = 1.01
1551 reflections
118 parameters
H-atom parameters constrained
Δρ_max_ = 0.32 e Å^−3^
Δρ_min_ = −0.26 e Å^−3^

### 

Data collection: *CAD-4 Software* (Enraf–Nonius, 1985 ▶); cell refinement: *CAD-4 Software*; data reduction: *XCAD4* (Harms & Wocadlo, 1995 ▶); program(s) used to solve structure: *SHELXS97* (Sheldrick, 2008 ▶); program(s) used to refine structure: *SHELXL97* (Sheldrick, 2008 ▶); molecular graphics: *SHELXTL* (Sheldrick, 2008 ▶); software used to prepare material for publication: *SHELXTL*.

## Supplementary Material

Crystal structure: contains datablocks I, global. DOI: 10.1107/S1600536808017728/ci2613sup1.cif

Structure factors: contains datablocks I. DOI: 10.1107/S1600536808017728/ci2613Isup2.hkl

Additional supplementary materials:  crystallographic information; 3D view; checkCIF report

## Figures and Tables

**Fig. 1.**
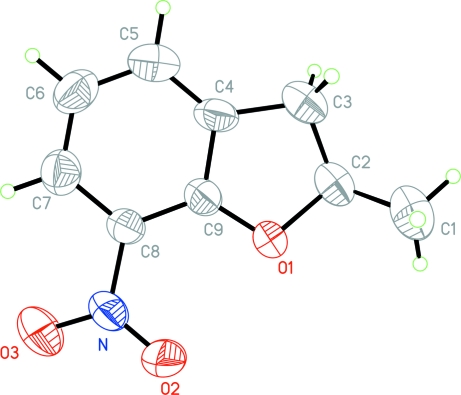
The molecular structure of the title compound, showing the atom-numbering scheme. Displacement ellipsoids are drawn at the 40% probability level.

**Fig. 2.**
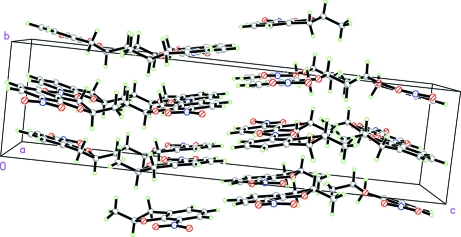
A view of the molecular packing in the title compound.

## supplementary crystallographic information

### Comment

The tittle compound, 2-methyl-7-nitro-2,3-dihydrobenzofuran, is an important intermediate for the synthesis of 2-methyl-2,3-dihydrobenzofuran-7-amine. we report here the crystal structure of the title compound.

The molecular structure of the compound is shown in Fig. 1. Bond lengths and angles are within normal ranges (Allen *et al.*, 1987), except the C1—C2 bond length of 1.420 (6) Å. The dihydrofuran ring is in an envelope conformation with C2 as flap atom. The nitro group is slightly twisted away from the attached benzene ring [O2—N—C8—C9 = 5.3 (5)° and O3—N—C8—C7 = 5.9 (5)°]. No hydrogen bonding interactions are observed in the crystal structure (Fig.2).

### Experimental

The title compound was synthesized according to the literature method (Majumdar *et al.*, 2008). Single crystals were obtained by slow evaporation of a methanol (25 ml) solution of the compound (0.30 g, 1.6 mmol) at room temperature for about 4 d.

### Refinement

H atoms were positioned geometrically [C-H = 0.93-0.98 Å] and constrained to ride on their parent atoms, with *U*_iso_(H) = *xU*_eq_(C), where *x* = 1.2 for aromatic and methylene H and 1.5 for methyl H atoms.

### Crystal data

**Table:**

C_9_H_9_NO_3_	*F*_000_ = 752
*M**_r_* = 179.17	*D*_x_ = 1.382 Mg m^−^^3^
Orthorhombic, *P**b**c**a*	Mo *K*α radiation λ = 0.71073 Å
Hall symbol: -P 2ac 2ab	Cell parameters from 25 reflections
*a* = 8.4250 (17) Å	θ = 10–13º
*b* = 7.2260 (14) Å	µ = 0.11 mm^−^^1^
*c* = 28.295 (6) Å	*T* = 298 (2) K
*V* = 1722.6 (6) Å^3^	Block, colourless
*Z* = 8	0.30 × 0.20 × 0.20 mm

### Data collection

**Table:**

Enraf–Nonius CAD-4 diffractometer	*R*_int_ = 0.048
Radiation source: fine-focus sealed tube	θ_max_ = 25.2º
Monochromator: graphite	θ_min_ = 1.4º
*T* = 298(2) K	*h* = 0→10
ω/2θ scans	*k* = −8→8
Absorption correction: ψ scan(North *et al.*, 1968)	*l* = 0→33
*T*_min_ = 0.969, *T*_max_ = 0.979	3 standard reflections
2977 measured reflections	every 200 reflections
1551 independent reflections	intensity decay: none
829 reflections with *I* > 2σ(*I*)	

### Refinement

**Table:**

Refinement on *F*^2^	Secondary atom site location: difference Fourier map
Least-squares matrix: full	Hydrogen site location: inferred from neighbouring sites
*R*[*F*^2^ > 2σ(*F*^2^)] = 0.066	H-atom parameters constrained
*wR*(*F*^2^) = 0.182	*w* = 1/[σ^2^(*F*_o_^2^) + (0.06*P*)^2^ + 1.5*P*] where *P* = (*F*_o_^2^ + 2*F*_c_^2^)/3
*S* = 1.01	(Δ/σ)_max_ = 0.001
1551 reflections	Δρ_max_ = 0.32 e Å^−^^3^
118 parameters	Δρ_min_ = −0.26 e Å^−^^3^
Primary atom site location: structure-invariant direct methods	Extinction correction: none

### Special details

**Table:**

Geometry. All e.s.d.'s (except the e.s.d. in the dihedral angle between two l.s. planes) are estimated using the full covariance matrix. The cell e.s.d.'s are taken into account individually in the estimation of e.s.d.'s in distances, angles and torsion angles; correlations between e.s.d.'s in cell parameters are only used when they are defined by crystal symmetry. An approximate (isotropic) treatment of cell e.s.d.'s is used for estimating e.s.d.'s involving l.s. planes.
Refinement. Refinement of *F*^2^ against ALL reflections. The weighted *R*-factor *wR* and goodness of fit *S* are based on *F*^2^, conventional *R*-factors *R* are based on *F*, with *F* set to zero for negative *F*^2^. The threshold expression of *F*^2^ > σ(*F*^2^) is used only for calculating *R*-factors(gt) *etc*. and is not relevant to the choice of reflections for refinement. *R*-factors based on *F*^2^ are statistically about twice as large as those based on *F*, and *R*- factors based on ALL data will be even larger.

### Fractional atomic coordinates and isotropic or equivalent isotropic displacement parameters (Å^2^)

**Table:**

	*x*	*y*	*z*	*U*_iso_*/*U*_eq_	
N	0.5181 (3)	0.0623 (5)	0.39917 (10)	0.0581 (8)	
O1	0.2624 (3)	0.0149 (4)	0.32787 (7)	0.0601 (8)	
C1	0.1077 (6)	0.0403 (8)	0.25775 (16)	0.0970 (16)	
H1A	0.1909	−0.0204	0.2405	0.146*	
H1B	0.1258	0.1715	0.2576	0.146*	
H1C	0.0074	0.0140	0.2431	0.146*	
O2	0.5612 (3)	0.0409 (5)	0.35839 (9)	0.0867 (10)	
C2	0.1064 (4)	−0.0249 (8)	0.30507 (14)	0.0812 (14)	
H2A	0.0947	−0.1598	0.3038	0.097*	
O3	0.6098 (3)	0.0689 (6)	0.43222 (10)	0.0992 (12)	
C3	−0.0172 (4)	0.0473 (6)	0.33963 (14)	0.0706 (11)	
H3A	−0.0614	0.1638	0.3289	0.085*	
H3B	−0.1025	−0.0413	0.3438	0.085*	
C4	0.0753 (4)	0.0725 (5)	0.38480 (12)	0.0532 (9)	
C5	0.0285 (4)	0.1112 (5)	0.42971 (13)	0.0634 (10)	
H5A	−0.0788	0.1240	0.4368	0.076*	
C6	0.1416 (5)	0.1314 (6)	0.46479 (13)	0.0624 (10)	
H6A	0.1093	0.1557	0.4956	0.075*	
C7	0.3000 (4)	0.1164 (5)	0.45498 (12)	0.0560 (9)	
H7A	0.3747	0.1319	0.4789	0.067*	
C8	0.3491 (4)	0.0774 (5)	0.40863 (11)	0.0460 (8)	
C9	0.2360 (4)	0.0537 (4)	0.37377 (11)	0.0459 (8)	

### Atomic displacement parameters (Å^2^)

**Table:**

	*U*^11^	*U*^22^	*U*^33^	*U*^12^	*U*^13^	*U*^23^
N	0.0376 (15)	0.085 (2)	0.0513 (17)	−0.0068 (16)	−0.0090 (16)	0.0044 (16)
O1	0.0358 (11)	0.104 (2)	0.0411 (12)	0.0006 (13)	−0.0058 (12)	−0.0061 (12)
C1	0.066 (3)	0.151 (5)	0.074 (3)	−0.004 (3)	−0.022 (3)	0.017 (3)
O2	0.0389 (13)	0.168 (3)	0.0530 (16)	0.0016 (17)	0.0045 (13)	−0.0016 (17)
C2	0.047 (2)	0.134 (4)	0.063 (2)	−0.004 (2)	−0.014 (2)	−0.002 (3)
O3	0.0446 (15)	0.176 (3)	0.0773 (19)	−0.0046 (19)	−0.0248 (16)	−0.009 (2)
C3	0.0399 (19)	0.098 (3)	0.074 (3)	−0.003 (2)	−0.009 (2)	−0.002 (2)
C4	0.0313 (16)	0.070 (2)	0.058 (2)	0.0012 (16)	0.0062 (16)	0.0053 (18)
C5	0.044 (2)	0.069 (3)	0.077 (3)	0.0068 (18)	0.009 (2)	0.004 (2)
C6	0.069 (2)	0.073 (2)	0.0458 (19)	0.006 (2)	0.011 (2)	−0.0021 (18)
C7	0.060 (2)	0.061 (2)	0.047 (2)	−0.0053 (19)	−0.0032 (18)	0.0004 (17)
C8	0.0352 (17)	0.060 (2)	0.0426 (18)	−0.0023 (15)	−0.0007 (15)	0.0044 (16)
C9	0.0375 (16)	0.0554 (19)	0.0447 (17)	−0.0043 (15)	−0.0028 (16)	0.0030 (16)

### Geometric parameters (Å, °)

**Table:**

N—O3	1.214 (4)	C3—H3A	0.97
N—O2	1.219 (3)	C3—H3B	0.97
N—C8	1.453 (4)	C4—C5	1.360 (5)
O1—C9	1.347 (4)	C4—C9	1.396 (4)
O1—C2	1.492 (4)	C5—C6	1.384 (5)
C1—C2	1.420 (6)	C5—H5A	0.93
C1—H1A	0.96	C6—C7	1.367 (5)
C1—H1B	0.96	C6—H6A	0.93
C1—H1C	0.96	C7—C8	1.404 (4)
C2—C3	1.520 (6)	C7—H7A	0.93
C2—H2A	0.98	C8—C9	1.382 (4)
C3—C4	1.508 (5)		
			
O3—N—O2	123.0 (3)	C2—C3—H3B	111.1
O3—N—C8	118.6 (3)	H3A—C3—H3B	109.0
O2—N—C8	118.4 (3)	C5—C4—C9	120.7 (3)
C9—O1—C2	108.2 (3)	C5—C4—C3	131.8 (3)
C2—C1—H1A	109.5	C9—C4—C3	107.5 (3)
C2—C1—H1B	109.5	C4—C5—C6	119.5 (3)
H1A—C1—H1B	109.5	C4—C5—H5A	120.3
C2—C1—H1C	109.5	C6—C5—H5A	120.3
H1A—C1—H1C	109.5	C7—C6—C5	121.2 (3)
H1B—C1—H1C	109.5	C7—C6—H6A	119.4
C1—C2—O1	109.7 (4)	C5—C6—H6A	119.4
C1—C2—C3	119.9 (4)	C6—C7—C8	119.6 (3)
O1—C2—C3	105.0 (3)	C6—C7—H7A	120.2
C1—C2—H2A	107.2	C8—C7—H7A	120.2
O1—C2—H2A	107.2	C9—C8—C7	119.2 (3)
C3—C2—H2A	107.2	C9—C8—N	122.3 (3)
C4—C3—C2	103.5 (3)	C7—C8—N	118.4 (3)
C4—C3—H3A	111.1	O1—C9—C8	126.9 (3)
C2—C3—H3A	111.1	O1—C9—C4	113.3 (3)
C4—C3—H3B	111.1	C8—C9—C4	119.8 (3)
			
C9—O1—C2—C1	145.4 (4)	O2—N—C8—C9	5.3 (5)
C9—O1—C2—C3	15.4 (4)	O3—N—C8—C7	5.9 (5)
C1—C2—C3—C4	−139.3 (4)	O2—N—C8—C7	−175.1 (3)
O1—C2—C3—C4	−15.5 (5)	C2—O1—C9—C8	172.0 (4)
C2—C3—C4—C5	−170.7 (4)	C2—O1—C9—C4	−8.9 (4)
C2—C3—C4—C9	10.9 (5)	C7—C8—C9—O1	−179.7 (3)
C9—C4—C5—C6	−0.2 (6)	N—C8—C9—O1	0.0 (6)
C3—C4—C5—C6	−178.4 (4)	C7—C8—C9—C4	1.3 (5)
C4—C5—C6—C7	1.2 (6)	N—C8—C9—C4	−179.1 (3)
C5—C6—C7—C8	−0.8 (6)	C5—C4—C9—O1	179.8 (3)
C6—C7—C8—C9	−0.4 (5)	C3—C4—C9—O1	−1.6 (4)
C6—C7—C8—N	180.0 (3)	C5—C4—C9—C8	−1.0 (6)
O3—N—C8—C9	−173.7 (3)	C3—C4—C9—C8	177.6 (3)
